# Characteristics of nursing home units with high versus low levels of person-centred care in relation to leadership, staff- resident- and facility factors: findings from SWENIS, a cross-sectional study in Sweden

**DOI:** 10.1186/s12877-021-02434-0

**Published:** 2021-09-16

**Authors:** Annica Backman, Per-Olof Sandman, Anders Sköldunger

**Affiliations:** 1 Department of Nursing, Umeå University, SE-901 87 Umeå, Sweden; 2 NVS, Division of Nursing, Karolinska Institutet, Huddinge, Sweden; 3 NVS, Division of Neurogeriatrics, Department of Nursing, Karolinska Institutet, Huddinge, Sweden

**Keywords:** Person centred care, Physical environment, Leadership, Nursing management, Nursing homes, Organisation of care

## Abstract

**Background:**

The context of care consists of factors that determines the extent to which staff can offer person-centred care. However, few studies have investigated factors that can explain variation in levels of person-centred care among nursing home units. The aim of this study was to explore factors characterizing nursing home units with high and low degree of person-centred care, with focus on leadership, staff, resident and facility factors.

**Methods:**

Cross-sectional data from residents, staff, and managers in 172 randomly selected nursing homes in Sweden were collected in 2014. Activities of Daily Living Index, Gottfries’ cognitive scale, Person-centred Care Assessment Tool together with demographic information and estimations of leadership engagement was used. Independent samples t-test and Chi2 test were conducted.

**Results:**

Highly person-centred units were characterised by leaders engaging in staff knowledge, professional development, team support and care quality. In highly person-centred units’ staff also received supervision of a nurse to a larger extent. Highly person-centred units were also characterised as dementia specific units, units with fewer beds and with a larger proportion of enrolled nurses. No differences in degree of person-centred care were seen between public or private providers.

**Conclusions:**

This study provides guidance for practitioners when designing, developing and adapting person-centred units in aged care contexts. Managers and leaders have an important role to promote the movement towards a person-centred practice of care, by supporting their staff in daily care, and engaging in staff knowledge and professional development. Targeting and adjusting environmental factors, such as provide small and dementia adapted environments to match the residents’ personal preferences and capacity are also important when striving towards person-centredness.

## Background

Moving away from task-oriented models of care, towards a more person-centred practice is now guiding the provision of care in nursing homes worldwide. The care context has been described as a decisive factor for the extent to which staff can provide person-centred care, but only a few studies have empirically investigated which factors define nursing home units as being more or less person-centred. To gain insight in determinants enhancing person-centred care with respect to personal factors (leadership, staffing and resident) as well as structural (facility) is essential.

The concept person-centred care is commonly used to indicate a holistic view of the person in need of care, respecting subjective experiences, values, needs and preferences [1–3]. Studies has shown that residents may benefit from this approach. Among persons living with dementia, it has been shown that person-centred care has been associated to higher quality of life [4, 5]. A person-centred intervention including staff education, environmental adaptation and a variety of daily activities for residents was shown to improve well-being among residents [6]. Also, Dementia Care Mapping (DCM) increased well-being among residents and reduced depressive symptoms in a person-centred intervention by Rokstad et al. [7]. A cluster-randomized trial showed that DCM and staff training in dementia care were associated with reduced agitation among nursing home residents [8]. However, opposite findings are also evident in previous literature as person-centred intervention studies with no or negative effect also has been reported. An individualised tailored intervention did not significantly change quality of life among residents [9] and an activity-oriented intervention showed no reduction in anxiety [10]. An increased number of falls accidents among residents were also reported from a person-centred intervention, while DCM reduced resident falls [8]. Positive impact on staff health and work situation has also been reported in previous literature. Working in a person-centred manner has been associated with higher degree of job satisfaction [11–14], higher psychosocial climate [13] lower degree of stress of conscience and lower degree of job strain [13, 15]. Taken together, available evidence indicates that person-centred care has predominantly beneficial outcomes wereas why person-centred practices have been declared by WHO as a global strategy to address the variety of care needs due to an aging population [1, 16] Although person-centred care is increasingly embraced and recommended by aged care providers, practitioners and the research society as the “gold standard” model of care [1, 10, 17, 18], there still remain challenges in putting a person-centred philosophy into practice [11].

Quality of leadership has been described as having potential to support or hinder person-centred practices [2, 19]. Until recently, a large amount of PCC intervention publications have, besides focusing on implementing PCC, and also highlighted the significance of leadership [20–23]. An intervention study using DCM have reported that managers who take an active part in the care practice, have clear visions, are supportive and act as role models with a leadership based on a person-centred philosophy, beneficially influenced the implementation of PCC in nursing homes [20]. Other studies have also reported that managers who promotes interpersonal relationships, communicating with staff with sensitivity, inclusion and respect affect implementation of a Person-Centred Care program [21] and a DCM intervention in a positive way [22]. One study showed that leadership is positively associated to degree of PCC in Swedish nursing homes [24]. Furthermore, staff in nursing home units in Sweden offering a high degree of PCC seem to be more satisfied with the leadership than units with low degree of PCC [25]. However, managerial obstacles have also been reported in previous studies where manager and leader resistance to change have been indicated as a barrier to enable person-centred practice [26]. A cluster randomized controlled trial of person-centred residential care by Chenoweth et al. [23] reported that the implementation effectiveness was low when the manager focused more on organisational efficiency than on enabling resident comfort and pleasure. In contrast, Jeon et al. [27] was not able to see any changes in terms of person-centred care after a 12-month leadership development programme, in their controlled trial. A systematic literature review concluded that leadership is a vital part of the implementation process in nursing, but research has still not specified in what way, and therefore suggests that more research is needed to explore the possible role of the leader [28]. Thus, although the quality of leadership has been described to have high potential to support or hinder person-centred practices [2, 19], there seems to be a limited consensus on leadership determinants enabling successful delivery of PCC.

The theoretical discourse has postulated that the organisational and contextual factors are critical for person-centred processes [2, 19]. A factor that has been pointed out as an important prerequisite for PCC, is staff competence [2]. A Canadian study showed that demographic characteristics of the staff (e.g., age, education, experience, job classification, ethnicity, work status) did not influence the provision of PCC, neither did facility characteristics (e.g., facility size, presence of a union, managers experience) [29]. In contrast, a Swedish study showed that units with high PCC had a more adapted environment and higher degree of staff educated for care of persons with dementia. Furthermore, staff in units with higher degree of PCC to a larger extent received regular supervision as well as reported satisfaction with leadership compared to units with lower degree of PCC [25]. In terms of other contextual factors such as ownership of the facility, it has been shown that public nursing homes has been related to higher quality in terms of higher staffing levels and offering individual accommodation/kitchen, but scored lower in terms of processual quality such as user participation, updated care plans and medication reviews, when comparing with private nursing homes [30].

Although the way leadership is performed can play an important role in PCC processes, there seems to be a lack of scientific consensus underpinning this assumption. Although the context of care delivery is increasingly recognised, organisational and contextual actors facilitating person-centred care are mainly based on theoretical models, with limited empirical knowledge on which factors actually characterise nursing home units offering different degree of PCC. Thus, it’s essential to gain insight in determinants enhancing person-centred care with respect to personal factors (leadership, staffing and resident) as well as structural (facility) therefor seems essential. The aim of this study was to explore factors characterizing high and low person-centred nursing home units, with focus on leadership, staff, resident and facility variables.

## Methods

### Design

The present study is part of the Swedish National Inventory of Care and Health in Residential Aged Care (SWENIS), a nationwide randomized longitudinal project with explorative design within The Umeå ageing and health research programme (U-AGE) [31]. The U-AGE research programme provides translational knowledge on the structure, content and outcomes of person-centred care and health-promoting living conditions in nursing homes for older people and people with dementia. The cross-sectional data for this study was collected between November 2013 and September 2014.

### Sampling

Out of 290 Swedish municipalities, a random selection of 60 municipalities was invited to participate in the project. Of the 60 municipalities 35 agreed to participate, contributing with data from 172 nursing homes. The final sample included data from staff *n* = 3605 (response rate 66.5%), residents *n* = 4831 (response rate 70%) and managers (*n* = 191). As this study is part of a large research programme [31], a more detailed description of sampling and data collection can found be in previous publications [24, 31].

### Data collection

Data was collected using a three-part survey that were sent out to the invited nursing homes. A self-reported staff survey comprising demographic information together with estimations of leadership engagement and person-centred care. All direct care staff working day/evening shifts with long-term employments were invited to participate. A resident survey collected demographic information about the residents together with assessments of the residents ADL and cognitive status. This survey was completed by the staff member who knew the resident best, their primary carer, through proxy-rating. Each primary carer commonly assessed one residents each. The third survey was an organisational survey consisting of questions about leadership and organisational characteristics about the nursing home. This part was completed by the managers.

### Characteristics of workforce and study context

In Sweden, nursing home managers have the operational responsibility for the care of residents, direct care staff and the work environment [32] and a qualification of social work or nursing care seems to be most common although no formal education is required to hold a managerial position in Sweden [33]. Registered nurses are responsible for the nursing care provision and medical care [34]. The direct care staff consists primarily of enrolled nurses and nurse assistants [34] and they are responsible for providing personal care and social services to residents [35]. Direct care staff consists primarily of enrolled nurses who have upper secondary level schooling (up to 3 years of training), with a level 4 qualification in the European Qualifications Framework [36] and nurses’ assistant who have approximately, half the length of training/education (< 1 year) with one level lower qualification in EQF [37–40]. The European Qualifications Framework is a translation device explicating qualification requirements within different educations and training systems in Europe [36]. Regular tasks for nurse assistants includes care assistance, making beds, helping patients with nutrition and hygiene, while enrolled nurses in addition also tests glucose, temperature, pulse, respiration and weight, carrying out simple changing bandages, conducting simple laboratory tests and giving medication on delegation from reg. nurse [39]. Swedish nursing homes are defined as housing for people 65 years and older, who are no longer able to live at home [41]. About 82,000 residents resides in nursing homes due to extensive personal care needs and/or cognitive impairment [37, 42]. The number of beds in municipal aged care in Sweden has decreased from 120,000 since 2000 although the proportion of older persons is growing and in 2014 the beds were 108,835 [43]. Swedish aged care is mainly funded publicly and essentially publicly produced, and the specification of the national policies postulates that older persons should have the possibility to live independently with high quality of life and furthermore that high-quality care should be provided to older persons in need of care [44].

### Study variables

Demographic data including staff characteristics (sex, age, qualifications, work experience) and resident characteristics (sex, age) as well as organisational characteristics of the facility (number of beds, SCU/general unit, private/public provider).

### Leader engagement and support

The extent to which staff perceived leader engagement and support were investigated by six study-specific single items inspired by Hällsten & Tengbland [45], and Beck [46],; *To what extent is your manager engaged in issues related to your knowledge/skills at work? To what extent is your managers engaged in issues related to your professional development at work? To what extent is your manager aware of the quality of the work you do? To what extent does your manager support you in providing care that is based on the individual older person’s needs? To what extent does your manager consciously work to improve the team spirit/mood of the staff group? To what extent do you get supervision of a reg. nurse in the direct care provision?* The items are rated on a five-point Likert scale, ranging from 1 (to a very small extent) to 5 (to a very large extent). Higher scores implied that staff to a larger extent agreed with the statement. The study-specific items were treated as single items in the analyses.

### Activities of daily living

Functional function of the residents was measured using a modified version of the Katz Activities of Daily Living Index (ADL) [47] previous published in K. Hulter Åsberg (1990) [48] which measures daily activities in six domains: eating, transferring, dressing, bathing, toileting, and continence. Each domain was scored dichotomously as dependent (0) or fully independent (1) to obtain a total score of 0–6 points. Higher score indicates a more independent functional function. Functional independence was defined as dependent or independent where dependence included person’s dependent in at least three of six items on the Katz ADL index [49].

### Gottfries’ cognitive scale (GCS)

Cognitive function was assessed using the scale developed by Gottfries and Gottfries [50], previously published in M. Gustafsson, U. Isaksson, S. Karlsson, PO. Sandman, H. Lovheim (2016). GCS consists of 27 items regarding ability to orientate. Statements are answered with a ‘yes’ (1 point) or ‘no’ (0 point). The range of the scale is 0–27 and high scores indicate a better orientation ability. Scores < 24 indicate cognitive impairment. Cut-off and criterion validity have been established against the Mini-Mental State Examination and been confirmed by Lövheim [51].

### Person-centred care assessment tool

The Swedish version of the Person-centred Care Assessment Tool (P-CAT) used to assess the extent to which the staff perceived care as being person-centred [52, 53]. P-CAT was developed by D. Edvardsson, D. Fetherstonhaugh, R. Nay and S. Gibson [52] and later translated to Swedish by K. Sjogren, M. Lindkvist, PO. Sandman, K. Zingmark and D. Edvardsson [53]. The Person-centred Care Assessment Tool includes 13 items rated on a five-point Likert scale, ranging from 1 (disagree completely) to 5 (agree completely). The total score is calculated with a possible range between 13 and 65 and higher scores indicate higher levels of person-centred care. As five items were negatively worded in P-CAT, (Items 7, 8, 9, 10, and 12) these were reversed before statistical analysis. Permission to use P-CAT was obtained from Professor D. Edvardsson. Free of charge.

### Data analyses

Data was analysed using SPSS statistics version 25. Normality was tested using Kolmogorov-Smirnov and visual examination of the histogram. Up to two missing items in the P-CAT instrument were replaced with the mean value of *the* individual for the total scale (< 8% of scale missing) [54]. A sample size calculation has been conducted for the SWENIS project and reported in previous literature [55, 56], indicating that a sample of 4500 residents would provide enough power to answer the U-Age SWENIS research questions at the 0.05 significance level. As a first step, staff-, resident- and facility characteristics were explored using descriptive statistics. Secondly, P-CAT was aggregated on unit level (mean value for the unit), and divided into two groups; units with higher mean values of P-CAT than the mean for all included nursing homes (49.78 points) and a second cohort including care units with a P-CAT score below the mean. As P-CAT was normally distributed a mean split was deemed appropriate. Thirdly, differences in leader engagement and support as well as staff-, resident- and facility characteristics between units with higher and lower levels of PCC were explored using independent samples t-test and Chi2 test.

## Results

Direct care staff consisted of mostly women (95.3%) with a mean age of 46.6 years (SD 11.3) and enrolled nursing was the most common qualification (82.5%). Direct care staff had 9.9 years (SD 8.0) as the average work experience in the nursing home. Nursing home managers (*n* = 191) were mostly women (91.0%) with a mean age of 49.6 years (SD 9.0) and they had been working as managers for approximately 3.4 years (SD 3.4) in that specific nursing home. Among managers, a social work degree was the most common educational qualification (47.9%) followed by registered nurse qualification (27.7%) and enrolled nursing (9.0%) qualifications. Approximately, 4.3% of the managers were human resource specialists and 11.2% had other qualifications. The sample of residents was comprised of mostly women (67.8%) and the mean age was 85.5 years. Their mean stay in the unit was about 30 months, and the majority were ADL dependent (84%) and cognitively impaired (66.6%) (see Tables 1 and 2). The participating nursing homes consisted of both regular units for older people (69.1%) and special care units for dementia (SCU) (30.9%). The number of beds varied between the nursing homes, 7–128 (mean 38) and most nursing homes were public (93.5%).

**Table 1:** Characteristics of staff (*n* = 3605) and managers (*n* = 191)

Staff	n ^1^(%)	m (SD)
Age (Years)		46.6 (11.3)
Sex		
Men	167 (4.7)	
Women	3401 (95.3)	
Qualifications		
Registered nurses	12 (0.3)	
Enrolled nurses	2918 (82,6)	
Nurse’s assistants	463 (13.1)	
No formal qualifications	82 (2.3)	
Other education	60 (1.7)	
Years of experience in aged care (mean ± SD)		17.9 (10.3)
Years in this nursing homes (mean (±SD)		9.9 (8.0)
Work shift		
Day shift	80 (2.2)	
Day and evening	3140 (88.2)	
Day, evening, night shift	318 (8.9)	
Managers	**n**^**2**^**(%)**	**m (SD)**
Age (Years; mean ± SD)		49.6 (9.0)
Sex		
Men	17 (9.0)	
Women	172 (91.0)	
Qualifications		
Registered nurses	52 (27.7)	
Enrolled nurses	17 (9.0)	
Social work	90 (47.9)	
Human resource specialist	8 (4.3)	
Other education	21 (11.2)	
Years of experience in aged care (mean ± SD)		11.0 (8.7)
Years of experience in this nursing home (mean ± SD)		3.4 (3.4)

^1^ n does not always add up to 3605 in all variables due to missing items varariables due to missing items

^2^ n does not always add up to 191 in all variables due to missing items

**Table 2:** Characteristics of residents (*n* = 4831)

	n ^1^ (%)	m (SD)
Age (Years)		85.5 (7.8)
Sex		
Men	1538 (32.2)	
Women	3239 (67.8)	
ADL Capacity		
Independent	716 (16)	
Dependent	3768 (84)	
Cognitive impairment		
Yes	2827 (66.6)	
No	1418 (33.4)	
Residing in SCU	1778 (37.8)	
Residing in general units	2931 (62.2)	
Length of stay in months (mean ± SD		30.5 (33.1)

^1^ n does not always add up to 4831 in all variables due to missing items

### Comparison of leader engagement and support in units with high and low scorers of PCC

When comparing how leader engagement and support were experienced by staff in units with higher and lower scoring of PCC, all variables were significantly higher in units scoring higher in PCC (see Table 3).

**Table 3:** Comparison of leadership engagement in units with high versus low degree of PCC

	Low degree of PCC		High degree of PCC		*p*-value
	n ^1^	m (SD)	n ^1^	m (SD)	
To what extent is your manager engaged in issues related to your knowledge / skills at work?	1817	3.3 (1.04)	1659	3.9 (0.95)	0.000
To what extent is your manager engaged in issues related to your professional development at work?	1820	3.3 (1.05)	1663	3.9 (0.97)	0.000
To what extent is your manager aware of the quality of the work you do?	1812	3.2 (1.11)	1661	3.8 (1.01)	0.000
To what extent does your manager support you in providing care that is based on the individual older person’s needs?	1816	3.4 (1.06)	1656	4.1 (0.96)	0.000
To what extent does your manager consciously work to improve the team spirit/mood of the staff group?	1815	3.1 (1.16)	1658	3.8 (1.07)	0.000
To what extent do you get supervision of a reg. Nurse in the direct care provision?	1820	3.3 (1.13)	1661	3.8 (1.05)	0.000

^1^ n = staff assessments. Does not always add up to 3605 staff all variables due to missing items

### Comparison of staff, resident and facility characteristics in units with high and low scores of PCC

There were no significant differences between staff in units with higher and lower scores of PCC in relation to age, sex and years of experience in aged care (see Table 4). In units with higher scores of PCC, a significantly higher proportion of staff (84.6%) were enrolled nurses compared to units with lower scores of PCC (80.7%) (*p* = < 0.002). Units with higher scores of PCC had a significantly lower proportion of nurse’s assistants (11.8%) compared to units with lower scores of PCC (14.2%) (*p* = 0.021). Units with higher scores of PCC had a significantly lower proportion of other education (1.7%) compared to units with lower scores of PCC (2.2%) (*p* = 0.016). In units with higher scores of PCC, staff work experience in current nursing home unit were significantly shorter (9.6 year), compared to units with lower scores of PCC (10.2 year) (*p* = 0.029) (see Table 4).

**Table 4:** Comparison of staff, resident and facility characteristics’ between units with high and low degree of PCC

	Low degree of PCC		High degree of PCC		*p*-value
	n ^1^ (%)	m (SD)	n ^1^ (%)	m (SD)	
**Staff**					
Age (Years)		47 (11.2)		46.2 (11.4)	0.051
Sex					
Women	1748 (95.6)		1600 (95.2)		
Men	81 (4.4)		81 (4.8)		0.582
Years of experience in aged care		18.3 (10.3)		17.6 (10.4)	0.074
Years in this nursing home unit		10.2 (8.2)		9.6 (7.8)	0.029
Education					
Nurse	5 (0.3)		7 (0.4)		0.327
Enrolled nurse	1468 (80.7)		1404 (84.6)		0.002
Nurse assistants	258 (14.2)		196 (11.8)		0.021
No formal education	47 (2.6)		33 (2.0)		0.144
Other	40 (2.2)		60 (1.7)		0.016
**Resident**					
Age		85.4 (7.9)		85.6 (7.8)	0.454
Sex					
Women	1428 (67.8)		1576 (67.4)		
Men	678 (32.2)		764 (32.6)		0.746
Length of stay (months)		31.1 (31.3)		30.2 (35.1)	0.460
Cognitive impairment	1281 (68.7)		1316 (63.1)		0.000
ADL dependent	1154 (49.1)		1195 (50.9)		0.008
**Facility**					
Number of beds per unit		13.4 (5.8)		12.4 (5.8)	> 0.001
Public nursing home^2^ assessmetns	1566 (94.6)		1439 (93.4)		
Private nursing home^2^	90 (5.4)		102 (6.6)		0.159
Dementia specific unit^2^	559 (30.6)		571 (34.3)		
General unit^2^	1269 (69.4)		1094 (65.7)		0.019

^1^ n does not always add up to 3605 staff or 4831 residents in all variables due to missing item

^2^ n=PCC assessments by staff

There were no significant differences in terms of resident age, sex and length of stay between units with higher and lower scores of PCC. When comparing facility variables, a significantly larger proportion of dementia specific units (34.3%) were found in highly scored PCC units, compared to the units with low scoring of PCC (30.6%) (*p* = 0.019). Units with higher scores of PCC had significantly higher proportion of residents with ADL dependency (50.9%) compared to units with lower scoring of PCC (49.1%) (see Table 4). It was also found that the number of beds per units were significantly lower 12.4 (SD 5.8) in units with high PCC scoring compared to in units with low scoring of PCC 13.4 (SD 5.8). No significant differences were found related to ownership in terms of public or privately-operated nursing homes (see Table 4).

## Discussion

This study aimed to explore factors characterizing high and low PCC nursing home units, with focus on leadership, staff, resident and facility variables. This study showed that leadership engagement and support were scored higher in highly person-centred units, which is in line with previous Swedish nursing home studies [24, 25]. More specifically, the findings showed that high PCC-units were characterised by having leaders engaging in staff knowledge, professional development and leaders supporting staff to provide care based on the individual older person’s needs. Furthermore, working to improve the team spirit as a leader, as well as being aware of the quality provided by staff were scored higher in high PCC units. Other studies have described that developing PCC requires leaders that acknowledge staffs’ unique competences and skills, are engaged in care practices as well as promotes team performance [57–60]. It has been shown that when staff feel supported, acknowledged and valued at work, optimal performance and commitment are likely to follow [57–60]. One interpretation of this study’s findings is, when staffs’ individual knowledge, competence and the quality of their work is acknowledged and supported by their leader, a person-centred approach can be nurtured, both among the individual staff member and within the team.

The results also show that staff in high PCC units scored that they received supervision of a registered nurse (RN) in the direct care provision to a larger extent that in units with low PCC. Escrig-Pinol, Corazzini, Blodgett, Chu, & McGilton [61], reports that effective nurse supervisors and support may improve work environments and staff’s ability to respond to residents’ needs in a timely, effective and compassionate manner. The expertise and clinical excellence a RN holds has shown to be crucial to build a person-centred approach [62]. Based on this, providing supervision to direct care staff appear to be an important focus of efforts when seeking to improve PCC.

The results also indicate that high PCC-units are characterised by a significantly higher proportion of staff with higher educational qualification. This has been elaborated in person-centred theory [2]. They postulate that a prerequisite is that staff have competence, being committed to the job, being able to demonstrate clarity of beliefs and values, and also knowledge and skills to make decisions’ and prioritise care [2]. In Sweden, the education of enrolled nurses is twice as long as nurse’s assistants’ education, hence it seems reasonable that a longer education may have contributed to a foundation of the principles necessary for PCC. However, this finding is contrary to a previous Canadian study [29], where level of education did not influence the provision of PCC and it was reported that individual factors such as education exerted very little influence on staffs’ ability to provide PCC, whereas access to resources and seemed to be more of a predictor. This study adds new insights on structural conditions of significance for PCC, as de facto the proportion of staff with enrolled nursing education was significantly higher in high PCC units. This finding contributes to the literature as staff competence has been highlighted as a critical element in person-centred theory [2, 19]. Staff work experience was significantly shorter in high PCC units compared to units with low degree of PCC. It is well known that cultural values of conservative traditions can maintain a strong influence over long periods of time, with resistance to change traditional care to a more person-oriented care as a consequence [63–65]. One can interpret that staff with shorter work experience more easily adapt to this person-centred culture shift, preferred by the national guidelines in Sweden [41] than staff with long experience of working with traditional care models. If this is the case here, subsequent studies need to be explored.

A larger proportion of dementia specific units were found in high PCC units, compared to units with low PCC. It was also found that the number of beds per units were lower in units with high PCC compared to in units with low PCC. This is consistent with previous findings from Swedish nursing home [25], where small, dementia-adapted environments characterised high PCC units in nursing homes. Previous research has reported that dementia-adapted environments may contribute to maintaining autonomy and independence and support social interactions and sense of self [66]. A reasonable implication is the need to tailor the physical environment to meet the individual needs of the residents and create small, homelike environments that allow the residents to be an active participant in everyday life rather than a passive recipient of care [66]. Swedish nursing home care has undergone a transformation under the last decade with a rapidly growing share of private actors, from 1% in 1990 to 16% in 2010 [67], to approximately 21% in 2016 [30]. The proponents argued that contracting nursing homes would lead the private actors to develop better ways to provide care with a quality improvement as a result [68, 69]. However, this study’s finding did not show any care quality differences in terms of PCC provision, related to the ownership of the nursing homes.

Previous intervention research from health care contexts [70], showed that a leadership emphasised PCC values and working practice, and interprofessional team working was facilitators’ for implementing person-centred care in hospital contexts. This can be situated as this study findings as highly person-centred units were characterised by leader engaging and support staff in providing a care that is based on the individual older person’s needs and works to improve the team spirit. This implies that this current study’s finding can be used as managerial strategies when designing and developing person-centred interventions in nursing homes care contexts as well. In summary, this study contributes to the existing research evidence, by suggesting that the support from managers and leaders is important for facilitating PCC, and highlights the importance of manager and leader support in daily care to enact staff to provide such care. However, further comparative, longitudinal and interventional studies would be valuable to confirm or reject these findings on leading towards PCC.

### Methodological considerations

This cross-sectional study cannot answer questions of causal nature. All resident data is proxy-rated which may introduce rate bias when rating items concerning one’s own work. This has been addressed with written information to the raters. Proxy-rated resident data may introduce recall and/or observer bias; still, it may be the best source of information due to the high prevalence of cognitive impairment in the sample, and the time the proxies had known the person they assessed was fairly long, indicating good knowledge about the older person. As this study draws on extensive cross-sectional randomised data from a national sample of staff and residents in Swedish nursing homes, this may serve as a means of avoiding systematic bias. The results are from a Swedish context, and thus, may affect the findings generalizability. However, as this study draws on extensive cross-sectional randomised data from a national sample of staff in Swedish nursing homes, it seems reasonable to argue that the findings could be applied across different contexts and settings with similar care structure. Differences between nursing homes were not explored, as this was not the aim of this study, still an important research area subsequent studies can explore.

## Conclusions and recommendations

This study provides information about leadership, staff, resident, facility determinants with capacity to enhancing person-centred care provision, were support from leaders seems important when striving towards person-centred care in daily practice. The study also highlights several environmental factors associated with highly person-centred units. The findings suggest that factors of leadership, staff, resident and facility can be identified and targeted in efforts to facilitate PCC practice in nursing home care. Addressing these gaps may provide important insight into the factors that help or hinder the provision and development PCC. The study findings can be interpreted as predictors or facilitators for PCC and be used for leadership training and/or development initiatives, or even as an empirical knowledge base for nursing curricula on nursing leadership and development. If nursing homes units or facilities struggles with implementing person-centred care, it seems that managers have an important role to promote the movement towards a person-centred practice of care, by supporting their staff in daily care, and engaging in staff knowledge and professional development. It also seems important to target and adjust environmental factors, such as provide small and dementia adapted environments to match the residents’ personal preferences and capacity. It also seems reasonable to suggest that educational initiatives need to be contextually embedded and tailored to meet the person in need of care when seeking to develop and improve person-centred nursing home units. This study provides guidance for practitioners when designing, developing and adapting person-centred units in aged care contexts.

## Data Availability

The datasets used and/or analyzed during the current study are available from the corresponding author on reasonable request.

## Abbreviations

PCC: Person-centred care
WHO: World Health Organization
ADL: Activities in daily living
RN: Registered nurse
